
JOURNAL INFORMATION
==============================
NLM Title Abbreviation: Natl Sci Rev
Journal ID: Natl Sci Rev
NLM Journal ID: 101633095
Journal ID: nsr

National Science Review

ISSN: 2095-5138
EISSN: 2053-714X

ARTICLE INFORMATION
==============================
PMCID: PMC7108005
PMID: 34676125
DOI: 10.1093/nsr/nwaa041
Article ID: nwaa041
Article version: 1
Subjects: Perspectives, AcademicSubjects/MED00010, AcademicSubjects/SCI00010

Pathogenic T cells and inflammatory monocytes incite inflammatory storm in severe COVID-19 patients

Zhou Yonggang
Fu Binqing
Zheng Xiaohu
Wang Dongsheng
Zhao Changcheng
qi Yingjie
Sun Rui
Tian Zhigang
Xu Xiaoling xxlahh8@ustc.edu.cn
Wei Haiming ustcwhm@ustc.edu.cn
1 Institute of Immunology and the CAS Key Laboratory of Innate Immunity and Chronic Disease, School of Life Science and Medical Center, University of Science and Technology of China , Hefei, Anhui 230001, China
2 Hefei National Laboratory for Physical Sciences at Microscale, University of Science and Technology of China , Hefei, Anhui 230001, China
3 The First Affiliated Hospital of USTC, Division of Life Sciences and Medicine, University of Science and Technology of China , Hefei, Anhui, 230001, China
Corresponding author. E-mail: ustcwhm@ustc.edu.cnCorresponding author. E-mail: xxlahh8@ustc.edu.cnThese authors contributed equally

Lead Contact

Electronic publication date: 2020 Mar 13
Electronic Location ID: nwaa041
Copyright: © The Author(s) 2020. Published by Oxford University Press on behalf of China Science Publishing & Media Ltd.
Copyright year: 2020
License: This is an Open Access article distributed under the terms of the Creative Commons Attribution License (http://creativecommons.org/licenses/by/4.0/), which permits unrestricted reuse, distribution, and reproduction in any medium, provided the original work is properly cited.
License URL: https://creativecommons.org/licenses/by/4.0/

edited-state: accepted-manuscript
article-lifecycle: PAP

==============================
**PERSPECTIVE**

**IMMUNOLOGY**

### Pathogenic T cells and inflammatory monocytes incite inflammatory storm in severe COVID-19 patients

Yonggang Zhou[1,2,3#], Binqing Fu[1,2,#], Xiaohu Zheng[1,2,#], Dongsheng Wang[3], Changcheng Zhao[3], Yingjie qi[3], Rui Sun[1,2], Zhigang Tian[1,2], Xiaoling Xu[3,*], Haiming Wei[1,2,4,*]

1. Institute of Immunology and the CAS Key Laboratory of Innate Immunity and Chronic Disease, School of Life Science and Medical Center, University of Science and Technology of China, Hefei, Anhui 230001, China

2. Hefei National Laboratory for Physical Sciences at Microscale, University of Science and Technology of China, Hefei, Anhui 230001, China

3. The First Affiliated Hospital of USTC, Division of Life Sciences and Medicine, University of Science and Technology of China, Hefei, Anhui, 230001, China

4. Lead Contact

#. These authors contributed equally

*. Correspondence: ustcwhm@ustc.edu.cn (H.W.); xxlahh8@ustc.edu.cn (X.X.)

Pathogenic human coronavirus infections, such as severe acute respiratory syndrome CoV (SARS-CoV) and Middle East respiratory syndrome CoV (MERS-CoV), cause high morbidity and mortality[1, 2]. Recently, a severe pneumonia-associated respiratory syndrome caused by a new coronavirus (SARS-CoV-2) was reported at December 2019 in the city Wuhan, Hubei province, China[3, 4, 5], which was also named as pneumonia-associated respiratory syndrome (PARS)[6] and can cause coronavirus disease 2019 (COVID-19) to seriously endanger human health. Up to 24th of February 2020, at least 77779 cases have been reported with 2666 fatal cases according to the report from China CDC. However, the immune mechanism that potential orchestrated acute mortality from COVID-19 patients is still unknown. Here we show that after the SARS-CoV-2 infection, $CD4^+$ T lymphocytes are rapidly activated to become pathogenic T helper (Th) 1 cells and generate GM-CSF etc. The cytokines environment induces inflammatory $CD14^+CD16^+$ monocytes with high expression of IL-6 and accelerate the inflammation. Given that large amount of inflammatory cells infiltrations have been observed in lungs from severe COVID-19 patients[7, 8], these aberrant pathogenic Th1 cells and inflammatory monocytes may enter the pulmonary circulation in huge numbers and play an immune damaging role to causing lung functional disability and quick mortality. Our results demonstrate that excessive non-effective host immune responses by pathogenic T cells and inflammatory monocytes may associate with severe lung pathology. Thus, we suggest that monoclonal antibodies targeting GM-CSF or interleukin 6 may be effective in blocking inflammatory storms and, therefore, be a promising treatment of severe COVID-19 patients.

Coronavirus, including SARS and MERS, has caused two large-scale pandemics in the last two decades[1, 2]. Although viral evasion of host immune responses and virus-induced cytopathic effects are believed to be critical in disease severity, studies from humans who died of SARS and animal models suggested that an excessive and aberrant host cytokine storm resulting in an exuberant immunopathology and lethal disease[9, 10, 11]. Inflammatory cytokine storm refers to the immune system gone awry and an excessive inflammatory response flaring out of control. Cytokine storms are associated with a wide variety of infectious and noninfectious diseases including graft-versus-host disease, autoimmune disease, severe virus infection, multiple organ dysfunction syndromes and chimeric antigen receptor (CAR)-T cell therapy[12, 13]. It has been reported that following SARS-CoV infection, dysregulated cytokine/chemokine responses and higher virus titers cause an inflammatory cytokine storm with lung immunopathological injury[12, 14]. Such Inflammation associated with the cytokine storm may begin at one local site but further spread throughout the body via the systemic circulation[12, 14]. Similarly, patients infected with SARS-CoV-2, that have been reported recently, have increased plasma concentrations of inflammation related cytokines, including interleukins (IL) 2, 7, and 10, granulocyte-colony stimulating factor (G-CSF), interferon-γ-inducible protein 10 (IP10), monocyte chemoattractant protein 1 (MCP1), macrophage inflammatory protein 1 alpha (MIP1A), and tumour necrosis factor α (TNF-α), especially in moribund patients[15]. Importantly, COVID-19 patients have developed characteristic pulmonary ground glass changes on imaging and peripheral lymphocytes decreasing[14, 16, 17]. More importantly, a large number of inflammatory immune cell infiltrations were also found in a COVID-19 patient with pulmonary pathology[7, 8]. These phenomena suggest severe pulmonary inflammation and cytokine storm also exist in SARS-CoV-2 infection. At present, symptomatic treatments with organ support to moribund patients are the mainstays of clinical managements[17]. It is urgent to identify the immunopathology mechanism to delay the pulmonary immune injury.

In patients infected with SARS-CoV, it has been reported that the severity of pulmonary immune injury correlated with extensive infiltration of neutrophils and macrophages in the lungs[18, 19], accompanied with increased numbers of neutrophils and monocytes and lower $CD8^+$ and $CD4^+$ T cell counts in the peripheral blood samples[20, 21, 22]. To identify the immune characteristic of patients infected with SARS-CoV-2, peripheral blood samples from patients with severe pneumonia were collected for immune analysis. Consistent with previous clinical characteristics reports[23], these hospitalized patients with confirmed SARS-CoV-2 infection involved from The First Affiliated Hospital of University of Science and Technology of China commonly have fever symptoms. The patients in intensive care unit (ICU) have significantly decreased concentrations of haemoglobin and albumin, but increased concentrations of C-reactive protein, alanine aminotransferase, aspartate aminotransferase and lactate dehydrogenase (Supplementary Tab. 1). The number of total leukocytes in peripheral blood had no significant differences between COVID-19 patients and healthy controls, whereas the number of lymphocytes decreased significantly in ICU patients. Specifically, monocytes from both ICU and non-ICU patients significantly decreased compared with healthy controls. The number of T cells also significantly decreased from both ICU and non-ICU patients. The

$CD4^+$ T cells from both patients in ICU and non-ICU decreased remarkably, whereas $CD8^+$ T cells decreased more significantly in ICU patients. Other kinds of leukocytes, including granulocyte, B cells and NK cells are not been significantly changed in numbers between COVID-19 patients and healthy controls (Supplementary Fig. 1).

To demonstrate the status of these aberrant altered T cells, several lymphoid antigens have been analyzed on T cells. These $CD4^+$ T cells in COVID-19 patients have higher expression of CD69, CD38, and CD44 compared with healthy controls (Supplementary Fig. 2A, B, C), indicating their activated status. OX40 have been reported to play a major role in promoting clonal expansion and inducing production of several cytokines in T cells[24]. In COVID-19 patients, OX40 expression increased remarkably on $CD4^+$ T cells, especially in severe ICU patients (Supplementary Fig. 2B, C). $CD8^+$ T cells in COVID-19 patients also showed activated phenotype with higher expression of CD69, CD38 and CD44 (Supplementary Fig. 2D, E). 41BB (CD137; TNFRS9) is an activation-induced co-stimulatory molecule, which is important to prime immune responses of cytotoxic $CD8^+$ T cells[25]. In ICU patients infected with SARS-CoV-2, the expression of 41BB increased significantly compared to healthy controls (Supplementary Fig. 2D, E). It has been reported that co-expression of Tim-3 and PD-1 may represent a subset of T cells with more severe exhaustion in virus infections[26, 27]. It is worth noting that much higher percentage of co-expression $Tim3^+PD\text{-}1^+$ T subset exist both in $CD4^+$ and $CD8^+$ T cells from COVID-19 patients (Supplementary Fig. 2F-I), especially in ICU patients, suggesting the exhausted status in T cells in these patients infected SARS-CoV-2.

To further identify the key pathogenic cytokines and the main source of these cytokines, interferon-γ (IFN-γ), TNF-α, granulocyte-macrophage colony-stimulating factor (GM-CSF) and IL-6 have been selected to be analyzed through intracellular cytokine staining, for these inflammatory mediators have been proven to be critical as the primary cause of inflammatory cytokine storm in patients infected with SARS-CoV or MERS-CoV[28, 29]. Without re-stimulation with PMA or incubation with monensin, high percentage of GM-CSF$^{+}$ and IL-6$^{+}$ expressions could be found in CD4$^{+}$ T cells from COVID-19 patients in both ICU and non-ICU patients compared to healthy controls (Fig. 1A, C). ICU patients with more severe pneumonia showed correlated higher percentage of GM-CSF$^{+}$ and IL-6$^{+}$CD4$^{+}$ T cells (Fig. 1A, C). Pathogenic Th1 cells with both IFN-γ and GM-CSF expression have been reported in central nervous system inflammation[30]. Importantly, aberrant pathogenic Th1 cells with co-expressing IFN-γ and GM-CSF exist only in ICU patients infected SARS-CoV-2, whereas little was found in non-ICU patients and healthy controls, indicating this pathogenic Th1 cells which have correlative evidence from patients with severe disease, play a critical role for hyper-inflammatory responses in SARS-CoV-2 pathogenesis (Fig. 1B, D). Meanwhile, TNF-α was not significantly up-regulated in CD4$^{+}$ T cells from COVID-19 patients (Supplementary Fig. 3A, B). CD8$^{+}$ T cells from ICU patients also showed higher expression of GM-CSF compared to those from non-ICU patients and healthy controls. IL-6 and TNF-α were not found in CD8$^{+}$ T cells (Supplementary Fig. 3C, D). Neither NK cells nor B cells were the secreting source of GM-CSF and IL-6 (Supplementary Fig. 3E-H).

GM-CSF has been recently involved in the pathogenesis of inflammatory and autoimmune diseases, in a mechanism that controls diverse pathogenic capabilities of inflammatory myeloid cells. Among these myeloid cells, monocyte is the pathogenic GM-CSF responsive cells that require GM-CSF to initiate tissue damage in both mouse and human[31,32]. To identify whether inflammatory monocyte exist in COVID-19 patients, phenotype and subpopulation of monocytes have been analysis. $CD14^+CD16^+$ inflammatory monocyte subsets seldom exist in healthy controls. By contrast, significantly higher percentage of $CD14^+CD16^+$ inflammatory monocyte exists in peripheral blood of COVID-19 patients. The percentage of $CD14^+CD16^+$ monocyte was much higher in severe pulmonary syndrome patients from ICU (Fig. 2A, C). Moreover, these monocyte from COVID-19 patients also showed capability to secrete GM-CSF. Importantly, significantly higher expression of IL-6 secreted from these inflammatory monocyte especially in ICU patients, which let the inflammatory storm even worse (Fig. 2B, D). Meanwhile, the number of $GM\text{-}CSF^+$ monocytes and $IL\text{-}6^+$ monocytes increased rapidly (Fig. 2E), suggesting the potential high risk of inflammatory cytokine storm caused by monocytes that may migrate to the lung and further develop into macrophage or monocyte derived dendritic cells. Thus, in COVID-19 patients, GM-CSF potentially links the severe pulmonary syndrome-initiating capacity of pathogenic Th1 cells ($GM\text{-}CSF^+IFN\text{-}\gamma^+$) with the inflammatory signature of monocytes ($CD14^+CD16^+$ with high expression of IL-6) and their progeny. These activated immune cells may enter the pulmonary circulation in large numbers and played an immune damaging role in severe pulmonary syndrome patients (Fig. 3).

The study provides the detailed immunopathology report on SARS-CoV-2, suggesting excessive activated immune response caused by pathogenic $GM\text{-}CSF^+$ Th1 cells and inflammatory $CD14^+CD16^+$ monocytes may connect pulmonary immunopathology leading to deleterious clinical manifestations and even acute mortality after SARS-CoV-2 infections. Consistent with the situation with SARS-CoV or MERS-CoV[14, 33], it is remarkable that children always experience mild-moderate clinical illness, elderly individuals exhibit worse outcomes after infection with SARS-CoV-2, further indicating that mature excessive immune response towards these pathogenic human coronavirus infections play a key role in inducing severe pulmonary syndrome and even organ failure. Specific new drugs targeted SARS-CoV-2 may take a long time to evaluate and develop. At this critical moment, several marketed drugs to target inflammatory storm and reduce immunopathology could be considered[34]. Tocilizumab, that can specifically bind both membrane bound IL-6 receptor and soluble IL-6 receptor and inhibit signal transduction, is the first IL-6 blocking antibody approved for marketing and have proved its safety and effectiveness in therapy for rheumatoid arthritis[35]. In order to verify whether targeted IL-6 receptor and inflammatory signals, may potentially be the right way to save severe COVID-19 patients, we further launched the clinical trial using Tocilizumab to block the interleukin 6 receptor (ChiCTR2000029765). These severe patients recruited right now have inspiring clinical results including quickly decreased temperature and respiratory function improved. Many urgent questions remain to be answered. Evidence from alveolar washing fluid and organs autopsy from COVID-19 patients are further needed to verify whether and how these aberrant pathogenic immune cells play a fatal immune damage to cause organ functional disability and mortality.

### MATERIALS AND METHODS

For details, see supplementary data.

### SUPPLEMENTARY DATA

Supplementary data are available at NSR online.

### Acknowledgements

This work was supported by the China National Center for Biotechnology Development (2020YFC0843800), Natural Science Foundation of China (81788101, 81922028), Youth Innovation Promotion Association of Chinese Academy of Sciences (Grant 2019442).

### Author contributions

Y.Z., B.F. and X.Z performed the experiments, analyzed and interpreted the data. D.W., C. Z., and Y.Q. helped to collect samples and information from patients. R.S. established techniques of FACS and interpreted the data. Z.T. provided strategic planning and interpreted some data. X.X supervised the clinical treatment of patients of 2019-CoV and helped with data interpretation. H.W. supervised the project, provided crucial ideas, and assisted with data interpretation. B.F. wrote the manuscript with H.W.

### Competing interests

The authors declare no competing interests.

### References

1. Drosten C, Gunther S, Preiser W, van der Werf S, Brodt HR, Becker S*, et al.* Identification of a novel coronavirus in patients with severe acute respiratory syndrome. *N Engl J Med* 2003, **348**(20)**:** 1967-1976.

2. Azhar EI, Hui DSC, Memish ZA, Drosten C, Zumla A. The Middle East Respiratory Syndrome (MERS). *Infect Dis Clin North Am* 2019, **33**(4)**:** 891-905.

3. Wang C, Horby PW, Hayden FG, Gao GF. A novel coronavirus outbreak of global health concern. *Lancet* 2020.

4. Wu F, Zhao S, Yu B, Chen YM, Wang W, Song ZG*, et al.* A new coronavirus associated with human respiratory disease in China. *Nature* 2020.

5. Zhou P, Yang XL, Wang XG, Hu B, Zhang L, Zhang W*, et al.* A pneumonia outbreak associated with a new coronavirus of probable bat origin. *Nature* 2020.

6. Jiang S, Xia S, Ying T, Lu L. A novel coronavirus (2019-nCoV) causing pneumonia-associated respiratory syndrome. *Cell Mol Immunol* 2020.

7. Xu Z, Shi L, Wang Y, Zhang J, Huang L, Zhang C*, et al.* Pathological findings of COVID-19 associated with acute respiratory distress syndrome. *The Lancet Respiratory Medicine*.

8. Tian S, Hu W, Niu L, Liu H, Xu H, Xiao S-Y. Pulmonary pathology of early phase 2019 novel coronavirus (COVID-19) pneumonia in two patients with lung cancer. *Journal of Thoracic Oncology* 2020.

9. Hui DSC, Zumla A. Severe Acute Respiratory Syndrome: Historical, Epidemiologic, and Clinical Features. *Infect Dis Clin North Am* 2019, **33**(4)**:** 869-889.

10. Rockx B, Baas T, Zornetzer GA, Haagmans B, Sheahan T, Frieman M*, et al.* Early upregulation of acute respiratory distress syndrome-associated cytokines promotes lethal disease in an aged-mouse model of severe acute respiratory syndrome coronavirus infection. *J Virol* 2009, **83**(14)**:** 7062-7074.

11. Smits SL, de Lang A, van den Brand JM, Leijten LM, van IWF, Eijkemans MJ*, et al.* Exacerbated innate host response to SARS-CoV in aged non-human primates. *PLoS Pathog* 2010, **6**(2)**:** e1000756.

12. Tisoncik JR, Korth MJ, Simmons CP, Farrar J, Martin TR, Katze MG. Into the eye of the cytokine storm. *Microbiology and molecular biology reviews : MMBR* 2012, **76**(1)**:** 16-32.

13. Giavridis T, van der Stegen SJC, Eyquem J, Hamieh M, Piersigilli A, Sadelain M. CAR T cell-induced cytokine release syndrome is mediated by macrophages and abated by IL-1

blockade. *Nature medicine* 2018, **24**(6)**:** 731-738.

14. Channappanavar R, Perlman S. Pathogenic human coronavirus infections: causes and consequences of cytokine storm and immunopathology. *Semin Immunopathol* 2017, **39**(5)**:** 529-539.

15. Huang C, Wang Y, Li X, Ren L, Zhao J, Hu Y*, et al.* Clinical features of patients infected with 2019 novel coronavirus in Wuhan, China. *Lancet* 2020.

16. Li G, Fan Y, Lai Y, Han T, Li Z, Zhou P*, et al.* Coronavirus infections and immune responses. *J Med Virol* 2020.

17. Guan W-j, Ni Z-y, Hu Y, Liang W-h, Ou C-q, He J-x*, et al.* Clinical Characteristics of Coronavirus Disease 2019 in China. *New England Journal of Medicine* 2020.

18. Gu J, Gong E, Zhang B, Zheng J, Gao Z, Zhong Y*, et al.* Multiple organ infection and the pathogenesis of SARS. *J Exp Med* 2005, **202**(3)**:** 415-424.

19. Nicholls JM, Poon LL, Lee KC, Ng WF, Lai ST, Leung CY*, et al.* Lung pathology of fatal severe acute respiratory syndrome. *Lancet* 2003, **361**(9371)**:** 1773-1778.

20. Cui W, Fan Y, Wu W, Zhang F, Wang JY, Ni AP. Expression of lymphocytes and lymphocyte subsets in patients with severe acute respiratory syndrome. *Clin Infect Dis* 2003, **37**(6)**:** 857-859.

21. Li T, Qiu Z, Zhang L, Han Y, He W, Liu Z*, et al.* Significant changes of peripheral T lymphocyte subsets in patients with severe acute respiratory syndrome. *J Infect Dis* 2004, **189**(4)**:** 648-651.

22. Wang YH, Lin AS, Chao TY, Lu SN, Liu JW, Chen SS*, et al.* A cluster of patients with severe acute respiratory syndrome in a chest ward in southern Taiwan. *Intensive Care Med* 2004, **30**(6)**:** 1228-1231.

23. Wang D, Hu B, Hu C, Zhu F, Liu X, Zhang J*, et al.* Clinical Characteristics of 138 Hospitalized Patients With 2019 Novel Coronavirus-Infected Pneumonia in Wuhan, China. *JAMA* 2020.

24. Croft M, So T, Duan W, Soroosh P. The significance of OX40 and OX40L to T-cell biology and immune disease. *Immunol Rev* 2009, **229**(1)**:** 173-191.

25. Laderach D, Movassagh M, Johnson A, Mittler RS, Galy A. 4-1BB co-stimulation enhances human CD8(+) T cell priming by augmenting the proliferation and survival of effector CD8(+) T cells. *Int Immunol* 2002, **14**(10)**:** 1155-1167.

26. Khaitan A, Unutmaz D. Revisiting immune exhaustion during HIV infection. *Curr HIV/AIDS Rep*

2011, **8**(1)**:** 4-11.

27. Jin HT, Anderson AC, Tan WG, West EE, Ha SJ, Araki K*, et al.* Cooperation of Tim-3 and PD-1 in CD8 T-cell exhaustion during chronic viral infection. *Proc Natl Acad Sci U S A* 2010, **107**(33)**:** 14733-14738.

28. Drosten C, Seilmaier M, Corman VM, Hartmann W, Scheible G, Sack S*, et al.* Clinical features and virological analysis of a case of Middle East respiratory syndrome coronavirus infection. *Lancet Infect Dis* 2013, **13**(9)**:** 745-751.

29. Lew TW, Kwek TK, Tai D, Earnest A, Loo S, Singh K*, et al.* Acute respiratory distress syndrome in critically ill patients with severe acute respiratory syndrome. *JAMA* 2003, **290**(3)**:** 374-380.

30. Stienne C, Michieletto MF, Benamar M, Carrie N, Bernard I, Nguyen XH*, et al.* Foxo3 Transcription Factor Drives Pathogenic T Helper 1 Differentiation by Inducing the Expression of Eomes. *Immunity* 2016, **45**(4)**:** 774-787.

31. Huang H, Wang S, Jiang T, Fan R, Zhang Z, Mu J*, et al.* High levels of circulating GM-CSF(+)CD4(+) T cells are predictive of poor outcomes in sepsis patients: a prospective cohort study. *Cell Mol Immunol* 2019, **16**(6)**:** 602-610.

32. Croxford AL, Lanzinger M, Hartmann FJ, Schreiner B, Mair F, Pelczar P*, et al.* The Cytokine GM-CSF Drives the Inflammatory Signature of CCR2+ Monocytes and Licenses Autoimmunity. *Immunity* 2015, **43**(3)**:** 502-514.

33. Assiri A, Al-Tawfiq JA, Al-Rabeeah AA, Al-Rabiah FA, Al-Hajjar S, Al-Barrak A*, et al.* Epidemiological, demographic, and clinical characteristics of 47 cases of Middle East respiratory syndrome coronavirus disease from Saudi Arabia: a descriptive study. *Lancet Infect Dis* 2013, **13**(9)**:** 752-761.

34. Zumla A, Hui DS, Azhar EI, Memish ZA, Maeurer M. Reducing mortality from 2019-nCoV: host-directed therapies should be an option. *The Lancet*.

35. Davies R, Choy E. Clinical experience of IL-6 blockade in rheumatic diseases - implications on IL-6 biology and disease pathogenesis. *Seminars in immunology* 2014, **26**(1)**:** 97-104.

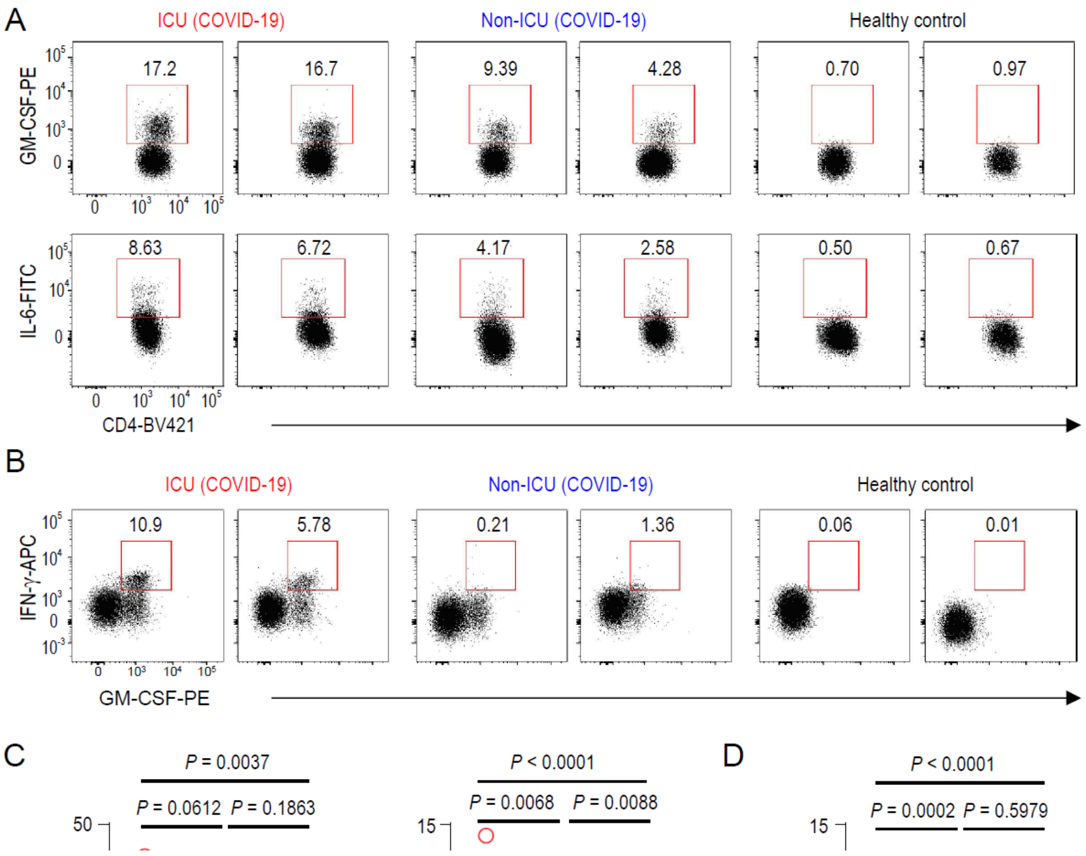

**Figure 1. Pathogenic Th1 cells with high expression of GM-CSF in COVID-19 patients.** (A) Representative density plots showing an analysis of GM-CSF and IL-6 expressions in gated CD45$^+$CD3$^+$CD4$^+$ T cells (Gating strategy showing in Supplementary Figure 2a) isolated from peripheral blood in healthy controls, ICU and non-ICU patients of COVID-19. (B) Representative density plots showing an analysis of co-expression of GM-CSF and IFN-γ in gated CD45$^+$CD3$^+$CD4$^+$ T cells isolated from peripheral blood in healthy controls, ICU and non-ICU patients of COVID-19. (C) Statistics calculated by the percentage of GM-CSF$^+$ or IL-6$^+$ cells from CD4$^+$ T cells. (D) Statistics calculated by the percentage of GM-CSF$^+$ and IFN-γ$^+$ co-expressing CD4$^+$ T cells. Data represent the mean ± SEM. One-way ANOVA. P<0.05 was considered statistically significant.

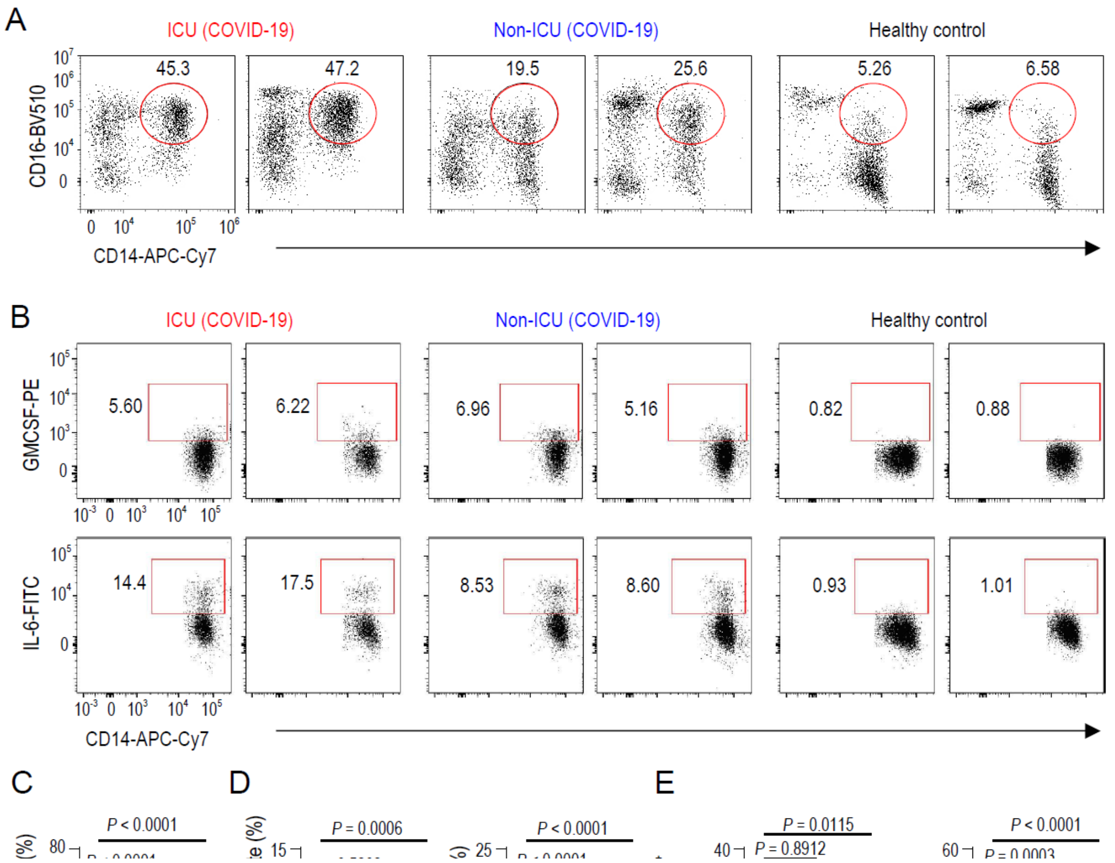

**Figure 2. Inflammatory monocytes with high expression of IL-6 in COVID-19 patients.** (A) Representative density plots showing an analysis of CD14 and CD16 expressions in gated CD45$^{+}$ monocytes (Gating strategy showing in Supplementary Figure 2a) isolated from peripheral blood in in healthy controls, ICU and non-ICU patients of COVID-19. (b) Representative density plots showing an analysis of GM-CSF and IL-6 expressions in gated CD45$^{+}$CD14$^{+}$ monocyte cells isolated from peripheral blood in healthy controls, in ICU and non-ICU patients of COVID-19. (c) Statistics calculated by the percentage of CD14$^{+}$CD16$^{+}$ subsets from monocytes. (d) Statistics calculated by the percentage of GM-CSF$^{+}$ or IL-6$^{+}$ cells from CD14$^{+}$ monocytes. (e) Statistics calculated by the cell number of GM-CSF$^{+}$ CD14$^{+}$ or IL-6$^{+}$CD14$^{+}$ monocytes. Data represent the mean ± SEM. One-way ANOVA. P<0.05 was considered statistically significant.

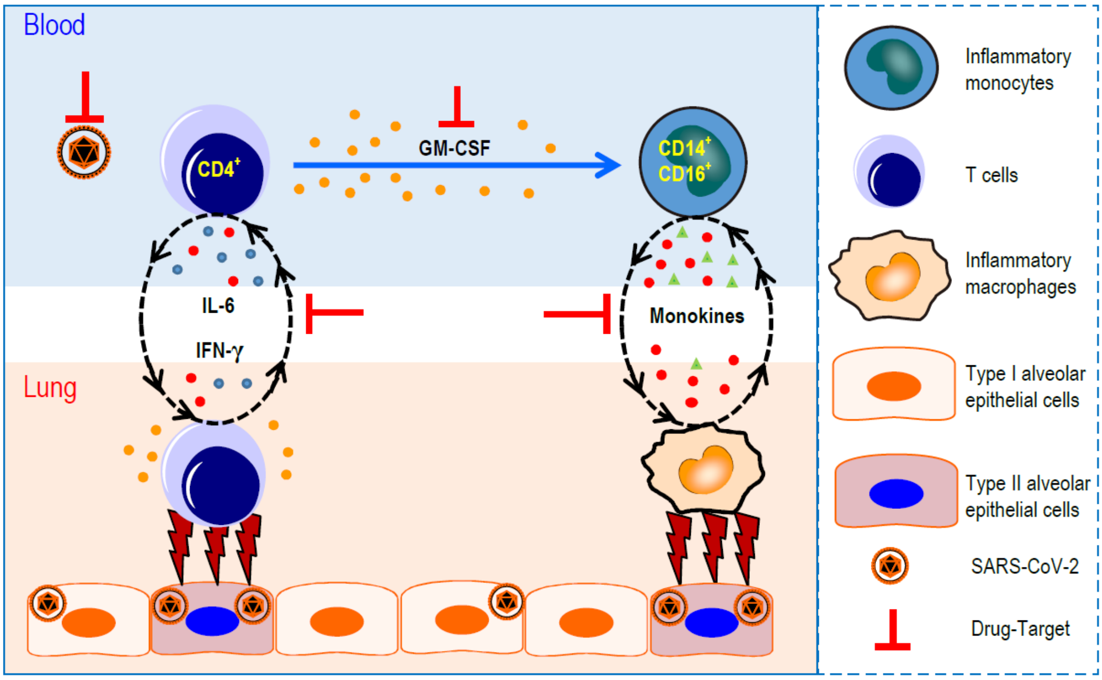

**Figure 3. Pathogenic Th1 cells and inflammatory monocytes in severe COVID-19.** Pathogenic $CD4^+$ Th1 ($GM\text{-}CSF^+IFN\text{-}\gamma^+$) cells were rapidly activated to produce GM-CSF and other inflammatory cytokines to form a cascade signature of inflammatory monocytes ($CD14^+CD16^+$ with high expression of IL-6) and their progeny. These activated immune cells may enter the pulmonary circulation in large numbers and played an immune damaging role in severe pulmonary syndrome patients. The monoclonal antibodies that targets the GM-CSF or interleukin 6 receptor may potentially prevent or curb immunopathology caused by COVID-19.
