# Pathogenic T-cells and inflammatory monocytes incite inflammatory storms in severe COVID-19 patients

**DOI:** 10.1093/nsr/nwaa041

**Published:** 2020-03-13

**Authors:** Yonggang Zhou, Binqing Fu, Xiaohu Zheng, Dongsheng Wang, Changcheng Zhao, Yingjie Qi, Rui Sun, Zhigang Tian, Xiaoling Xu, Haiming Wei

**Affiliations:** Institute of Immunology and the CAS Key Laboratory of Innate Immunity and Chronic Disease, School of Life Science and Medical Center, University of Science and Technology of China, China; Hefei National Laboratory for Physical Sciences at Microscale, University of Science and Technology of China, China; The First Affiliated Hospital of USTC, Division of Life Sciences and Medicine, University of Science and Technology of China, China; Institute of Immunology and the CAS Key Laboratory of Innate Immunity and Chronic Disease, School of Life Science and Medical Center, University of Science and Technology of China, China; Hefei National Laboratory for Physical Sciences at Microscale, University of Science and Technology of China, China; Institute of Immunology and the CAS Key Laboratory of Innate Immunity and Chronic Disease, School of Life Science and Medical Center, University of Science and Technology of China, China; Hefei National Laboratory for Physical Sciences at Microscale, University of Science and Technology of China, China; The First Affiliated Hospital of USTC, Division of Life Sciences and Medicine, University of Science and Technology of China, China; The First Affiliated Hospital of USTC, Division of Life Sciences and Medicine, University of Science and Technology of China, China; The First Affiliated Hospital of USTC, Division of Life Sciences and Medicine, University of Science and Technology of China, China; Institute of Immunology and the CAS Key Laboratory of Innate Immunity and Chronic Disease, School of Life Science and Medical Center, University of Science and Technology of China, China; Hefei National Laboratory for Physical Sciences at Microscale, University of Science and Technology of China, China; Institute of Immunology and the CAS Key Laboratory of Innate Immunity and Chronic Disease, School of Life Science and Medical Center, University of Science and Technology of China, China; Hefei National Laboratory for Physical Sciences at Microscale, University of Science and Technology of China, China; The First Affiliated Hospital of USTC, Division of Life Sciences and Medicine, University of Science and Technology of China, China; Institute of Immunology and the CAS Key Laboratory of Innate Immunity and Chronic Disease, School of Life Science and Medical Center, University of Science and Technology of China, China; Hefei National Laboratory for Physical Sciences at Microscale, University of Science and Technology of China, China

Pathogenic human coronavirus infections, such as severe acute respiratory syndrome CoV (SARS-CoV) and Middle East respiratory syndrome CoV (MERS-CoV), cause high morbidity and mortality [1,2]. Recently, a severe pneumonia-associated respiratory syndrome caused by a new coronavirus (SARS-CoV-2) was reported in December 2019 in the city of Wuhan, Hubei province, China [3–5], which was also named as pneumonia-associated respiratory syndrome (PARS) [6] and can cause coronavirus disease 2019 (COVID-19) to seriously endanger human health. Up to 24 February 2020, at least 77 779 cases had been reported, with 2666 fatal cases according to the report from China CDC. However, the immune mechanism that potentially orchestrates acute mortality from COVID-19 patients is still unknown. Here, we show that, after the SARS-CoV-2 infection, CD4^+^ T lymphocytes are rapidly activated to become pathogenic T helper (Th) 1 cells and generate GM-CSF, etc. The cytokine environment induces inflammatory CD14^+^CD16^+^ monocytes with a high expression of IL-6 and accelerates the inflammation. Given that a large number of inflammatory-cell infiltrations have been observed in lungs from severe COVID-19 patients [7,8], these aberrant pathogenic Th1 cells and inflammatory monocytes may enter the pulmonary circulation in huge numbers and play an immune-damaging role causing lung functional disability and quick mortality. Our results demonstrate that excessive non-effective host-immune responses by pathogenic T-cells and inflammatory monocytes may be associated with severe lung pathology. Thus, we suggest that monoclonal antibodies targeting GM-CSF or interleukin 6 may be effective in blocking inflammatory storms and, therefore, be a promising treatment for severe COVID-19 patients.

Coronavirus, including SARS and MERS, has caused two large-scale pandemics in the last two decades [1,2]. Although viral evasion of host-immune responses and virus-induced cytopathic effects are believed to be critical in disease severity, studies from humans who died of SARS and animal models have suggested that an excessive and aberrant host-cytokine storm results in an exuberant immunopathology and lethal disease [9–11]. Inflammatory cytokine storm refers to the immune system gone awry and an excessive inflammatory response flaring out of control. Cytokine storms are associated with a wide variety of infectious and non-infectious diseases including graft-versus-host disease, autoimmune disease, severe virus infection, multiple organ dysfunction syndromes and chimeric antigen receptor (CAR) T-cell therapy [12,13]. It has been reported that, following SARS-CoV infection, dysregulated cytokine/chemokine responses and higher virus titers cause an inflammatory cytokine storm with lung immunopathological injury [12,14]. Such inflammation associated with the cytokine storm may begin at one local site but spread farther throughout the body via the systemic circulation [12,14]. Similarly, patients infected with SARS-CoV-2 who have been reported recently have had increased plasma concentrations of inflammation-related cytokines, including interleukins (IL) 2, 7 and 10, granulocyte-colony-stimulating factor (G-CSF), interferon-γ-inducible protein 10 (IP10), monocyte chemoattractant protein 1 (MCP1), macrophage inflammatory protein 1 alpha (MIP1A) and tumour necrosis factor α (TNF-α), especially in moribund patients [15]. Importantly, COVID-19 patients have developed characteristic pulmonary ground glass changes on imaging and peripheral lymphocytes decreasing [14,16,17]. More importantly, a large number of inflammatory immune-cell infiltrations were also found in a COVID-19 patient with pulmonary pathology [7,8]. These phenomena suggest that severe pulmonary inflammation and cytokine storm also exist in SARS-CoV-2 infection. At present, symptomatic treatments with organ support to moribund patients are the mainstays of clinical management [17]. It is urgent to identify the immunopathology mechanism to delay the pulmonary immune injury.

In patients infected with SARS-CoV, it has been reported that the severity of pulmonary immune injury correlated with extensive infiltration of neutrophils and macrophages in the lungs [18,19], accompanied by increased numbers of neutrophils and monocytes and lower CD8^+^ and CD4^+^ T-cell counts in the peripheral blood samples [20–22]. To identify the immune characteristics of patients infected with SARS-CoV-2, peripheral blood samples from patients with severe pneumonia were collected for immune analysis. Consistently with previous clinical-characteristics reports [23], these hospitalized patients with confirmed SARS-CoV-2 infection involved in the First Affiliated Hospital of University of Science and Technology of China commonly have fever symptoms. Patients in intensive care units (ICUs) have significantly decreased concentrations of haemoglobin and albumin, but increased concentrations of C-reactive protein, alanine aminotransferase, aspartate aminotransferase and lactate dehydrogenase (Supplementary Table 1). The number of total leukocytes in peripheral blood had no significant differences between COVID-19 patients and healthy controls, whereas the number of lymphocytes had decreased significantly in ICU patients. Specifically, monocytes from both ICU and non-ICU patients significantly decreased compared with those of healthy controls. The number of T-cells also significantly decreased from both ICU and non-ICU patients. The CD4^+^ T-cells from both ICU and non-ICU patients had decreased remarkably, whereas CD8^+^ T-cells decreased more significantly in ICU patients. Other kinds of leukocytes, including granulocyte, B-cells and NK cells, had not significantly changed in numbers between COVID-19 patients and healthy controls (Supplementary Fig. 1).

To demonstrate the status of these aberrant altered T-cells, several lymphoid antigens have been analysed on T-cells. These CD4^+^ T-cells in COVID-19 patients have higher expression of CD69, CD38 and CD44 compared with healthy controls (Supplementary Fig. 2A–C), indicating their activated status. OX40 have been reported to play a major role in promoting clonal expansion and inducing the production of several cytokines in T-cells [24]. In COVID-19 patients, OX40 expression had increased remarkably on CD4^+^ T-cells, especially in severe ICU patients (Supplementary Fig. 2B and C). CD8^+^ T-cells in COVID-19 patients also showed activated phenotypes with higher expression of CD69, CD38 and CD44 (Supplementary Fig. 2D and E). 41BB (CD137; TNFRS9) is an activation-induced co-stimulatory molecule, which is important for the prime immune responses of cytotoxic CD8^+^ T-cells [25]. In ICU patients infected with SARS-CoV-2, the expression of 41BB had increased significantly compared to healthy controls (Supplementary Fig. 2D and E). It has been reported that co-expression of Tim-3 and PD-1 may represent a subset of T-cells with more severe exhaustion in virus infections [26,27]. It is worth noting that a much higher percentage of co-expression Tim3^+^PD-1^+^ T subsets existed in both CD4^+^ and CD8^+^ T-cells from COVID-19 patients (Supplementary Fig. 2F–I), especially in ICU patients, suggesting an exhausted status in T-cells in these patients infected with SARS-CoV-2.

To further identify the key pathogenic cytokines and the main source of these cytokines, interferon-γ (IFN-γ), TNF-α, granulocyte-macrophage co-lony- stimulating factor (GM-CSF) and IL-6 have been selected to be analysed through intracellular cytokine staining, for these inflammatory mediators have been proven to be critical as the primary cause of inflammatory cytokine storms in patients infected with SARS-CoV or MERS-CoV [28,29]. Without restimulation with PMA or incubation with monensin, a high percentage of GM-CSF^+^ and IL-6^+^ expressions could be found in CD4^+^ T-cells from COVID-19 patients in both ICU and non-ICU patients compared to healthy controls (Fig. 1A and C). ICU patients with more severe pneumonia showed a correlated higher percentage of GM-CSF^+^ and IL-6^+^CD4^+^ T-cells (Fig. 1A and C). Pathogenic Th1 cells with both IFN-γ and GM-CSF expression have been reported in central-nervous-system inflammation [30]. Importantly, aberrant pathogenic Th1 cells with co-expressing IFN-γ and GM-CSF existed only in ICU patients infected with SARS-CoV-2, whereas little was found in non-ICU patients and healthy controls, indicating that pathogenic Th1 cells, with correlative evidence from patients with severe disease, play a critical role in hyper-inflammatory responses in SARS-CoV-2 pathogenesis (Fig. 1B and D). Meanwhile, TNF-α was not significantly upregulated in CD4^+^ T-cells from COVID-19 patients (Supplementary Fig. 3A and B). CD8^+^ T-cells from ICU patients also showed a higher expression of GM-CSF compared to those from non-ICU patients and healthy controls. IL-6 and TNF-α were not found in CD8^+^ T-cells (Supplementary Fig. 3C and D). Neither NK cells nor B-cells were the secreting source of GM-CSF and IL-6 (Supplementary Fig. 3E–H).

**Figure 1.**
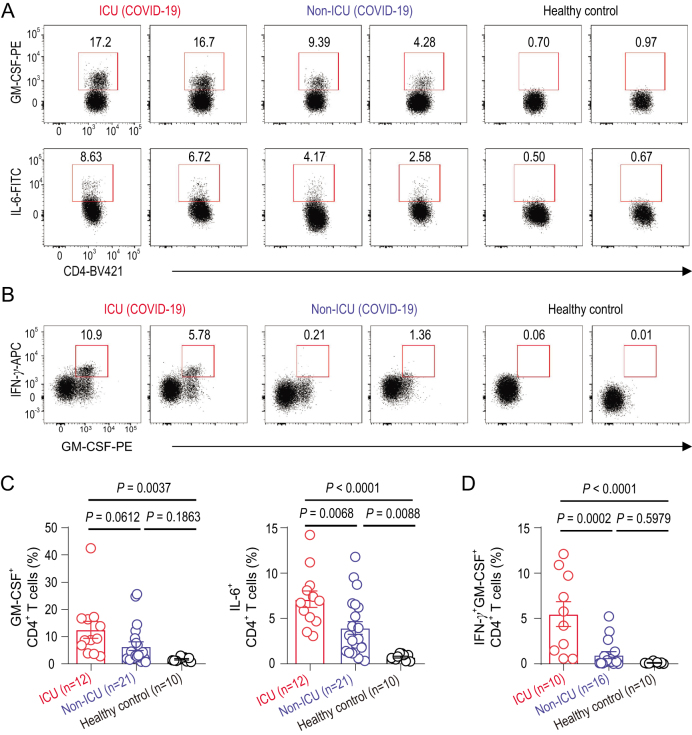
Pathogenic Th1 cells with high expression of GM-CSF in COVID-19 patients. (A) Representative density plots showing an analysis of GM-CSF and IL-6 expressions in gated CD45^+^CD3^+^CD4^+^ T-cells (gating strategy shown in Supplementary Fig. 2a) isolated from peripheral blood in healthy controls, ICU and non-ICU patients of COVID-19. (B) Representative density plots showing an analysis of co-expression of GM-CSF and IFN-γ in gated CD45^+^CD3^+^CD4^+^ T-cells isolated from peripheral blood in healthy controls, ICU and non-ICU patients of COVID-19. (C) Statistics calculated by the percentage of GM-CSF^+^ or IL-6^+^ cells from CD4^+^ T-cells. (D) Statistics calculated by the percentage of GM-CSF^+^ and IFN-γ^+^ co-expressing CD4^+^ T-cells. Data represent the mean ± SEM. One-way ANOVA. *P* < 0.05 was considered statistically significant.

GM-CSF has been recently involved in the pathogenesis of inflammatory and autoimmune diseases, in a mechanism that controls the diverse pathogenic capabilities of inflammatory myeloid cells. Among these myeloid cells, the monocyte is the pathogenic GM-CSF-responsive cells that require GM-CSF to initiate tissue damage in both mouse and human [31,32]. To identify whether inflammatory monocytes exist in COVID-19 patients, phenotypes and subpopulations of monocytes have been analysed. CD14^+^CD16^+^ inflammatory monocyte subsets seldom exist in healthy controls. By contrast, a significantly higher percentage of CD14^+^CD16^+^ inflammatory monocytes existed in the peripheral blood of COVID-19 patients. The percentage of CD14^+^CD16^+^ monocyte was much higher in severe-pulmonary-syndrome patients from the ICU (Fig. 2A and C). Moreover, these monocytes from COVID-19 patients also showed the capability to secrete GM-CSF. Importantly, a significantly higher expression of IL-6 was secreted from these inflammatory monocytes especially in ICU patients, which made the inflammatory storm even worse (Fig. 2B and D). Meanwhile, the number of GM-CSF^+^ monocytes and IL-6^+^ monocytes increased rapidly (Fig. 2E), suggesting the potentially high risk of inflammatory cytokine storms caused by monocytes that may migrate to the lung and further develop into macrophage- or monocyte-derived dendritic cells. Thus, in COVID-19 patients, GM-CSF potentially links the severe-pulmonary-syndrome-initiating capacity of pathogenic Th1 cells (GM-CSF^+^IFN-γ^+^) with the inflammatory signature of monocytes (CD14^+^CD16^+^ with high expression of IL-6) and their progeny. These activated immune cells may enter the pulmonary circulation in large numbers and played an immune-damaging role in severe-pulmonary-syndrome patients (Fig. 3).

**Figure 2.**
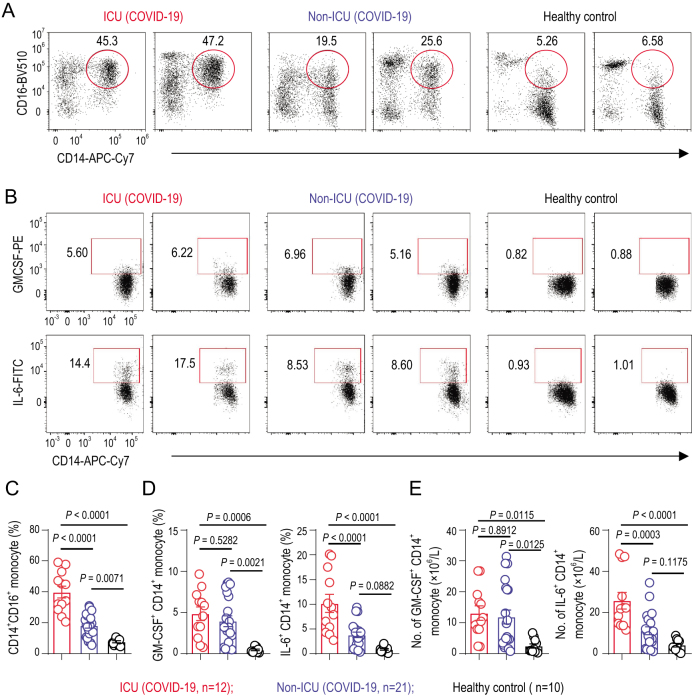
Inflammatory monocytes with high expression of IL-6 in COVID-19 patients. (A) Representative density plots showing an analysis of CD14 and CD16 expressions in gated CD45^+^ monocytes (gating strategy shown in Supplementary Fig. 2a) isolated from peripheral blood in healthy controls, ICU and non-ICU patients of COVID-19. (B) Representative density plots showing an analysis of GM-CSF and IL-6 expressions in gated CD45^+^CD14^+^ monocyte cells isolated from peripheral blood in healthy controls, ICU and non-ICU patients of COVID-19. (C) Statistics calculated by the percentage of CD14^+^CD16^+^ subsets from monocytes. (D) Statistics calculated by the percentage of GM-CSF^+^ or IL-6^+^ cells from CD14^+^ monocytes. (E) Statistics calculated by the cell number of GM-CSF^+^ CD14^+^ or IL-6^+^CD14^+^ monocytes. Data represent the mean ± SEM. One-way ANOVA. *P* < 0.05 was considered statistically significant.

**Figure 3.**
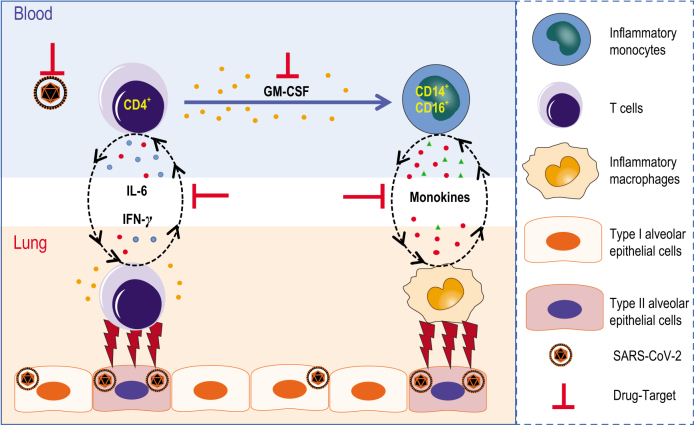
Pathogenic Th1 cells and inflammatory monocytes in severe COVID-19. Pathogenic CD4^+^ Th1 (GM-CSF^+^IFN-γ^+^) cells were rapidly activated to produce GM-CSF and other inflammatory cytokines to form a cascade signature of inflammatory monocytes (CD14^+^CD16^+^ with high expression of IL-6) and their progeny. These activated immune cells may enter the pulmonary circulation in large numbers and played an immune-damaging role in severe-pulmonary-syndrome patients. The monoclonal antibodies that target the GM-CSF or interleukin-6 receptor may potentially prevent or curb immunopathology caused by COVID-19.

The study provides a detailed immunopathology report on SARS-CoV-2, suggesting that the excessive activated immune response caused by pathogenic GM-CSF^+^ Th1 cells and inflammatory CD14^+^CD16^+^ monocytes may cause pulmonary immunopathology leading to deleterious clinical manifestations and even acute mortality after SARS-CoV-2 infections. Consistently with the situation with SARS-CoV or MERS-CoV [14,33], it is remarkable that children always experience mild–moderate clinical illness, whereas elderly individuals exhibit worse outcomes after infection with SARS-CoV-2, further indicating that mature excessive immune responses towards these pathogenic human coronavirus infections play a key role in inducing severe pulmonary syndrome and even organ failure. Specific new drugs targeting SARS-CoV-2 may take a long time to evaluate and develop. At this critical moment, several marketed drugs for targeting inflammatory storms and reducing immunopathology could be considered [34]. Tocilizumab, which can specifically bind both membrane-bound IL-6 receptors and soluble IL-6 receptors and inhibit signal transduction, is the first IL-6-blocking antibody approved for marketing and has proven its safety and effectiveness in therapy for rheumatoid arthritis [35]. In order to verify whether targeting IL-6 receptors and inflammatory signals may potentially be the right way to save severe COVID-19 patients, we further launched a clinical trial using Tocilizumab to block the IL-6 receptor (ChiCTR2000029765). Those severe patients who have been recruited so far have had inspiring clinical results, including quickly decreased temperatures and respiratory-function improvement. Many urgent questions remain to be answered. Evidence from alveolar washing fluid and organ autopsies from COVID-19 patients are further needed to verify whether and how these aberrant pathogenic immune cells play a part in fatal immune damage to cause organ functional disability and mortality.

## Supplementary Material

nwaa041_Supplemental_File
