# Supplementary material for: Pathogenic T-cells and inflammatory monocytes incite inflammatory storms in severe COVID-19 patients
Source: Natl Sci Rev. 2020 Mar 13;7(6):998–1002. doi: 10.1093/nsr/nwaa041 (PMC7108005; doi:10.1093/nsr/nwaa041)
Supplement: nwaa041_Supplemental_File [file nwaa041_supplemental_file.pdf]

SUPPLEMENTARY DATA

MATERIALS AND METHODS

Sample collection

Peripheral blood samples of patients infected with 2019-nCoV were collected from The First Affiliated Hospital of University of Science and Technology China (Hefei, Anhui) with the consents from all patients. All of the patients are hospitalized patients with confirmed infection of 2019-nCoV. The diagnosis of pneumonia of unknown cause was first based on clinical characteristics, chest imaging, and ruling out of other pneumonia related bacterial and viral pathogens. Suspected patients were isolated using airborne precautions in the designated hospital. A new coronavirus, which was named 2019-nCoV, was analyzed from lower respiratory tract specimens from these suspected patients. Patients who admitted to the ICU care because they required high-flow nasal cannula or higher-level oxygen support measures to correct hypoxaemia. The detailed patient information has been shown in **Supplementary Table 1**. Peripheral blood samples from Healthy controls were collected from the First Affiliated Hospital of Anhui Medical University, Hefei, Anhui, China. Ethical approval (No. 2020-XG (H)-005) was conducted in accordance with the international conference on Hamonisation Guidelines for good clinical practice and the Declaration of Helsinki and institutional ethics guidelines and obtained from the Ethics Committee from The First Affiliated Hospital of University of Science & Technology of China for emerging infectious diseases.

Flow cytometry

Suspensions of lymphocytes were stained for the following human monoclonal antibodies (**Supplementary Table 2**). Homologous IgGs were used as negative control antibodies. FACS staining was performed according to the manufacturer's instructions. Data from 50,000-80,000 single-cell events were collected using a standard Novocyte3130 flow cytometer (ACEA Biosciences). Intracellular staining of inflammatory cytokines were performed without adding any re-stimulation. The cells were then collected, washed, and blocked according to instructions of eBioscience (Foxp3 / Transcription Factor Staining Buffer Set, eBioscience). The antibodies used for intracellular staining are also shown in **Supplementary Table 2**.

Additional information

Correspondence and requests for materials should be addressed to Haiming Wei (ustcwhm@ustc.edu.cn)

Supplementary Figure 1. The altered composition of white blood cells in COVID-19 patients.

Quantification of leukocyte, monocyte, granulocyte, lymphocyte, NK cells, B cells, $CD4^+$ and $CD8^+$ T cells from flow cytometry analysis in COVID-19. Each dot represents one samples. Data represent the mean ± SEM. Data were analyzed by one-way ANOVA. P<0.05 was considered statistically significant.

Supplementary Figure 2. Activated T cells in COVID-19 patients.

(A) Gating strategy for monocyte, lymphocytes, $CD3^+$ T cells, $CD19^+$ B cells, $CD56^+$ NK cells, $CD4^+$ T cells and $CD8^+$ T cells. (B, C) Representative density plots and MFI statistics calculated for CD69, CD38, CD44 and OX40 expressions in gated $CD45^+CD3^+CD4^+$ T cells isolated from peripheral blood in healthy controls, ICU and non-ICU patients of COVID-19. (D, E) Representative density plots and MFI statistics calculated for CD69, CD38, CD44 and 41BB expressions in gated $CD45^+CD3^+CD8^+$ T cells isolated from peripheral blood in healthy controls, ICU and non-ICU patients of COVID-19. (F, G) Representative density plots and percentage statistics calculated for Tim-3 and PD-1 co-expressions in gated $CD45^+CD3^+CD4^+$ T cells isolated from peripheral blood in healthy controls, ICU and non-ICU patients of COVID-19. (H, I) Representative density plots and percentage statistics calculated for Tim-3 and PD-1 co-expressions in gated $CD45^+CD3^+CD8^+$ T cells isolated from peripheral blood in healthy controls, ICU and non-ICU patients of COVID-19. Data represent the mean ± SEM. One-way ANOVA. P<0.05 was considered statistically significant.

Supplementary Figure 3. The inflammatory cytokines expression characteristic in T cells, NK cells and B cells from COVID-19 patients.

(A, B) Representative density plots and percentage statistics calculated for TNF-α expressions in gated $CD45^+CD3^+CD4^+$ T cells isolated from peripheral blood in healthy controls, ICU and non-ICU patients of COVID-19. (C, D) Representative density plots and percentage statistics calculated for GM-CSF, IL-6 and TNF-α expressions in gated $CD45^+CD3^+CD8^+$ T cells isolated from peripheral blood in healthy controls, ICU and non-ICU patients of COVID-19. (E, F) Representative density plots and percentage statistics calculated for GM-CSF, IL-6 and TNF-α expressions in gated $CD45^+CD3^-CD56^+$ NK cells isolated from peripheral blood in healthy controls, ICU and non-ICU patients of COVID-19. (G, H) Representative density plots and percentage statistics calculated for GM-CSF and IL-6 expressions in gated $CD45^+CD3^+CD19^+$ B cells isolated from peripheral blood in healthy controls, ICU and non-ICU patients of COVID-19. Each dot represents one samples. Data represent the mean ± SEM. Data were analyzed by one-way ANOVA. P<0.05 was considered statistically significant.

Supplementary Table 1. Baseline characteristics and laboratory findings of COVID-19 patients in this study

Supplementary Table 2. Antibody list

Supplementary Figure. 1

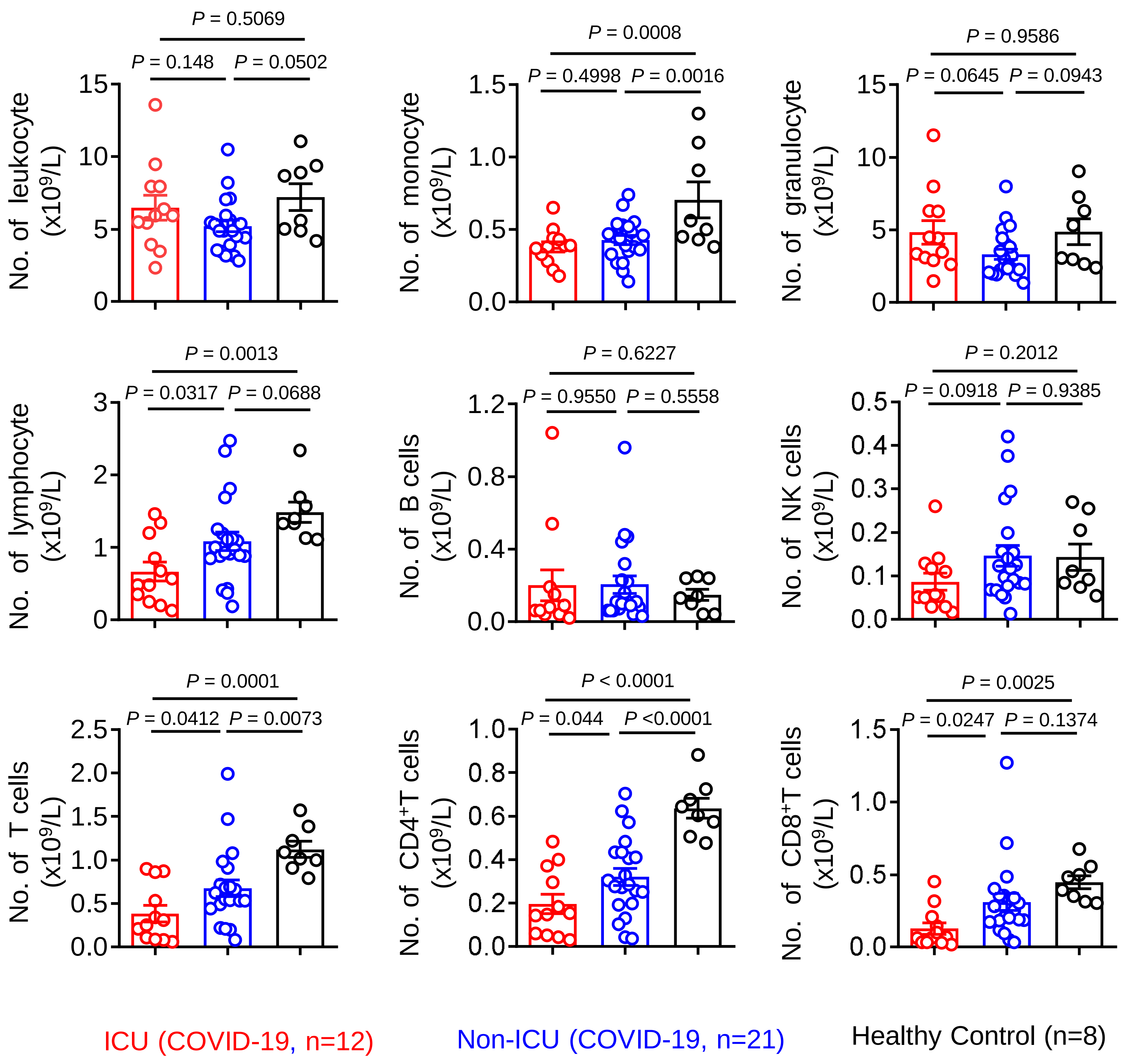

Supplementary Figure. 2

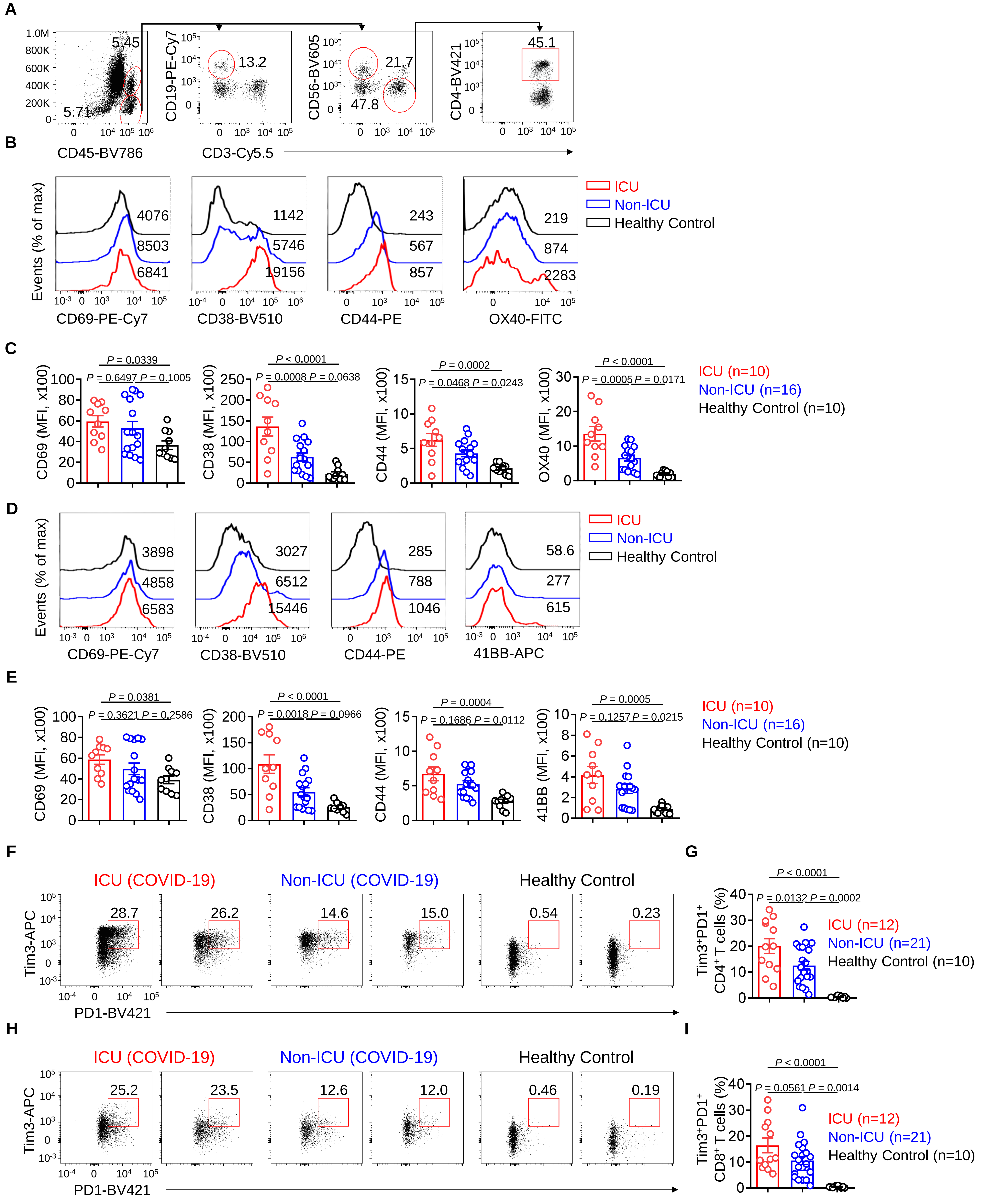

Supplementary Figure 3.

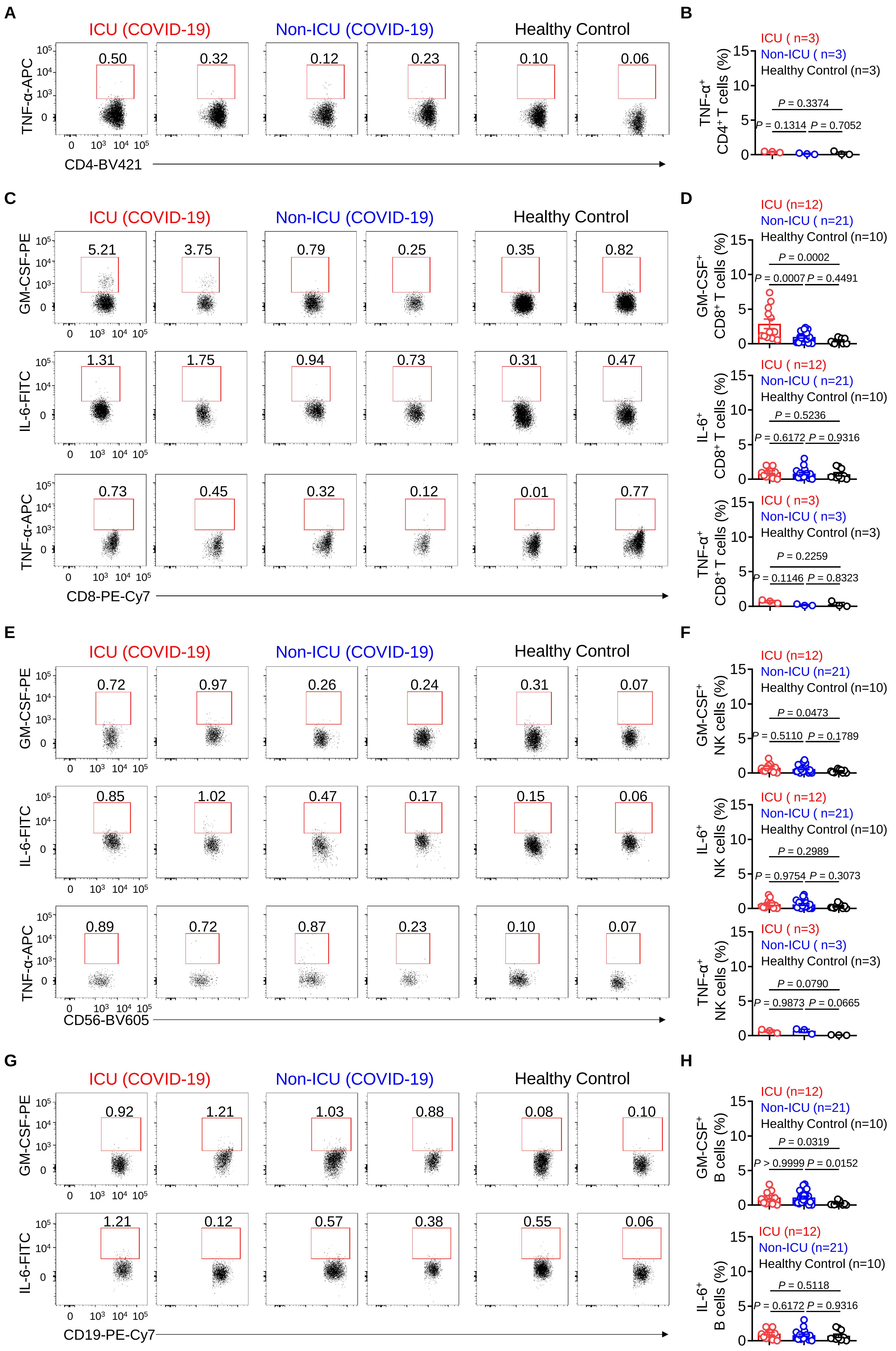

Supplementary Table 1. Baseline characteristics and laboratory findings of COVID-19 patients in this study

	ICU care (n=12)	No ICU care (n=21)	P value
Age, median(IQR), years	50(29-68)	41.8 (22-76)	0.12
Gender(M/F), No.(%)	9(75%)/3(25%)	13(61.9%)/8(38.1%)	0.46
Symptomes, fever, No.(%)	12(100%)	21 (100%)	
White blood cell count, $X10^9/L$, Median (IQR)	6.29 (2.32-13.58)	5.17 (2.82-10.49)	0.23
Neutrophil count, $X10^9/L$, Median (IQR)	4.98 (1.79-12.24)	3.36 (1.37-10.03)	0.10
Monocyte count, $X10^9/L$, Median (IQR)	0.36 (0.11-0.52)	0.33 (0.17-0.65)	0.63
Lymphocyte count, $X10^9/L$, Median (IQR)	0.86 (0.32-1.69)	1.33 (0.28-2.86)	0.04
Haemoglobin (g/l), Median (IQR)	125.42 (97-144)	145.24 111-162)	0.002
Platelet count, $X10^9/L$, Median (IQR)	194.58 (72-307)	184.88 (131-292)	0.64
C-reactive protein (mg/L), Median (IQR)	75.13 (8.7-175.2)	13.15 (0.5-91.1)	0.0002
Activated partial thromboplastin time, s, Median (IQR)	36.32 (27.9-42.9)	41.67 (24.4-67.8)	0.09
Prothrombin time, s, Median (IQR)	14.23 (12.7-15.3)	21.08 (12.1-142)	0.46
D-dimer, mg/L, Median (IQR)	0.61 (0.08-3.93)	0.17 (0.02-0.52)	0.09
Albumin, g/L, Median (IQR)	37.08 (29.9-47.6)	44.09 (37.3-53.6)	0.0015
Alanine aminotransferase, U/L, Median (IQR)	43 (16-95)	25 (12-15)	0.0021
Aspartate aminotransferase, U/L, Median (IQR)	40.25 (19-70)	24.71 (13-44)	0.0014
Total bilirubin, mmol/L, Median (IQR)	21.27 (7.9-53.8)	18.05 (8.8-37.5)	0.45
Potassium, mmol/L, Median (IQR)	3.85 (3.03-4.71)	4.07 (3.43-4.81)	0.22
Sodium, mmol/L, Median (IQR)	136.75 (133-142)	138.88 (131-146)	0.12
Creatinine, μmol/L, Median (IQR)	72.75 (54-96)	74.47 (54-101)	0.76
Creatine kinase, IU/L, Median (IQR)	220.46 (49.6-1011.4)	108.39 (31.3-273.9)	0.19
Lactate dehydrogenase, U/L, Median (IQR)	325.82 (189-513)	219.63 (117-338)	0.0021
Hypersensitive troponin I, pg/mL, Median (IQR)	0.08 (0.03-0.12)	0.07 (0.04-0.1)	0.11
Procalcitonin, ng/mL, Median (IQR)	0.19 (0.1-0.37)	0.19 (0.1-0.22)	0.47

Abbreviations: ICU, intensive care unit; IQR, interquartile range; P values indicate differences between ICU and non-ICU patients. $P<0.05$ was considered statistically significant. P values comparing ICU care and no ICU care are from Fisher's exact test or unpaired T test.

Supplementary Table 2. Antibody list

REAGENT	SOURCE	IDENTIFIER
Anti-human CD3 PerCP-CY5.5	Biolegend	Cat#300328
Anti-human CD14 APC-CY7	BD Pharmingen	Cat#557831
Anti-human CD16 BV510	Biolegend	Cat#563830
Anti-human CD56 BV605	Biolegend	Cat#362538
Anti-human CD45 BV786	Biolegend	Cat#563716
Anti-human CD4 BV421	Biolegend	Cat#562424
Anti-human CD8 PE-CY7	BD Pharmingen	Cat#557746
Anti-human CD19 PE-CY7	BD Pharmingen	Cat#557835
Anti-human IL6 FITC	BD Pharmingen	Cat#340526
Anti-human GM-CSF PE	BD Pharmingen	Cat#554507
Anti-human IFN-γ APC	BD Pharmingen	Cat#551385
Anti-human TNFα APC	BD Pharmingen	Cat#551384
Anti-human CD69 PE-CY7	BD Pharmingen	Cat#557745
Anti-human OX40 FITC	BD Pharmingen	Cat#555837
Anti-human 41BB APC	BD Pharmingen	Cat#550890
Anti-human CD44 PE	BD Pharmingen	Cat#555479
Anti-human CD38 BV510	BD Pharmingen	Cat#563251
Anti-human PD1 BV421	BD Pharmingen	Cat#562516
Anti-human Tim3 APC	BD Pharmingen	Cat#565558
Mouse IgG2a, κ FITC	BD Pharmingen	Cat#555573
Mouse IgG2b, κ FITC	BD Pharmingen	Cat#555742
Mouse IgG1, κ PE	BD Pharmingen	Cat#555749
Mouse IgG2a, κ PE	BD Pharmingen	Cat#555574
Mouse IgG2a APC	BD Pharmingen	Cat#555576
